# Supplementary figures and images for: Multi-omics analyses and machine learning prediction of oviductal responses in the presence of gametes and embryos
Source: eLife. 2025 Feb 26;13:RP100705. doi: 10.7554/eLife.100705 (PMC11864756; doi:10.7554/eLife.100705)

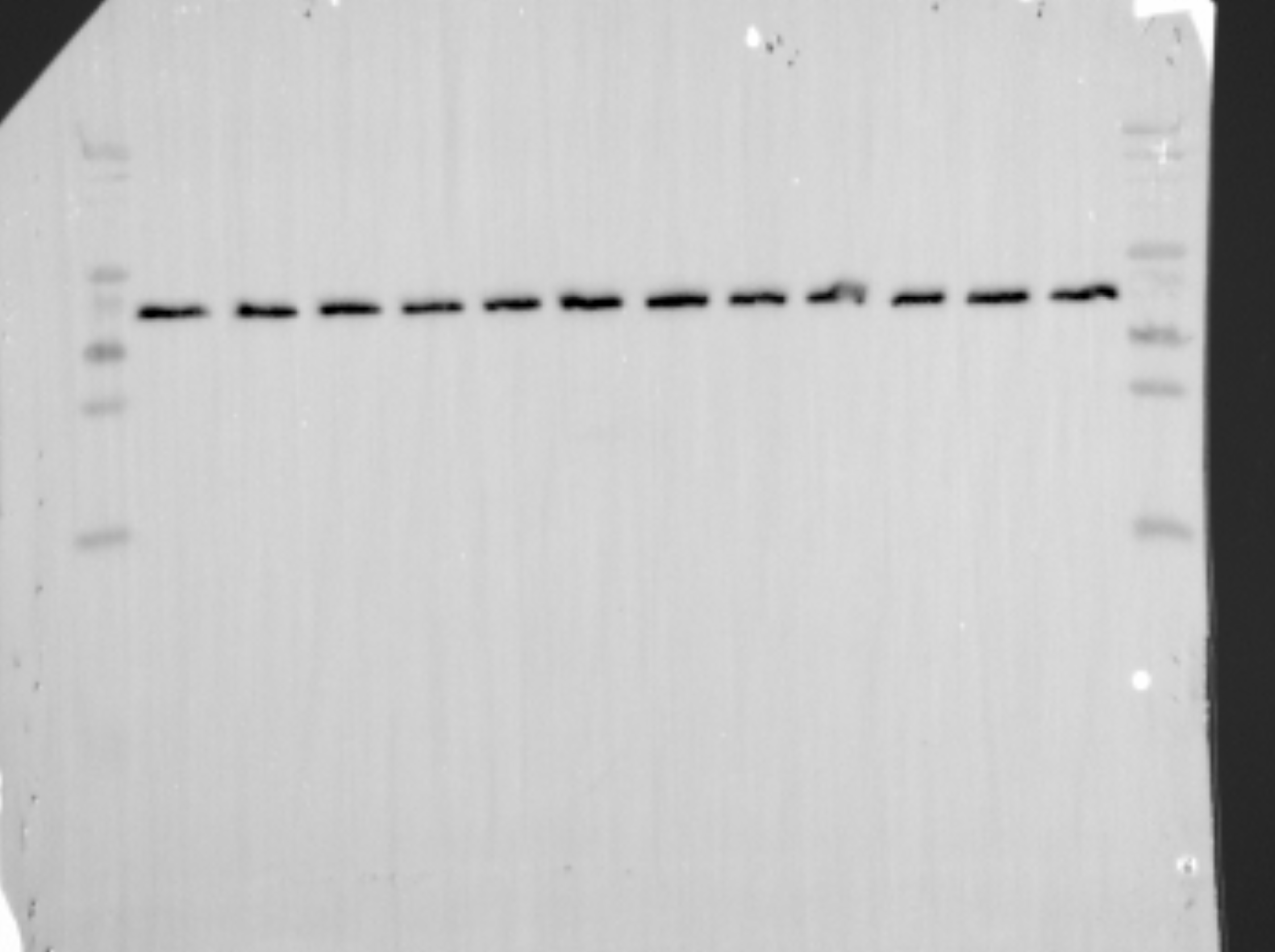

Supplement: Figure 3—source data 1. [file elife-100705-fig3-data1.zip › P38 total.png]

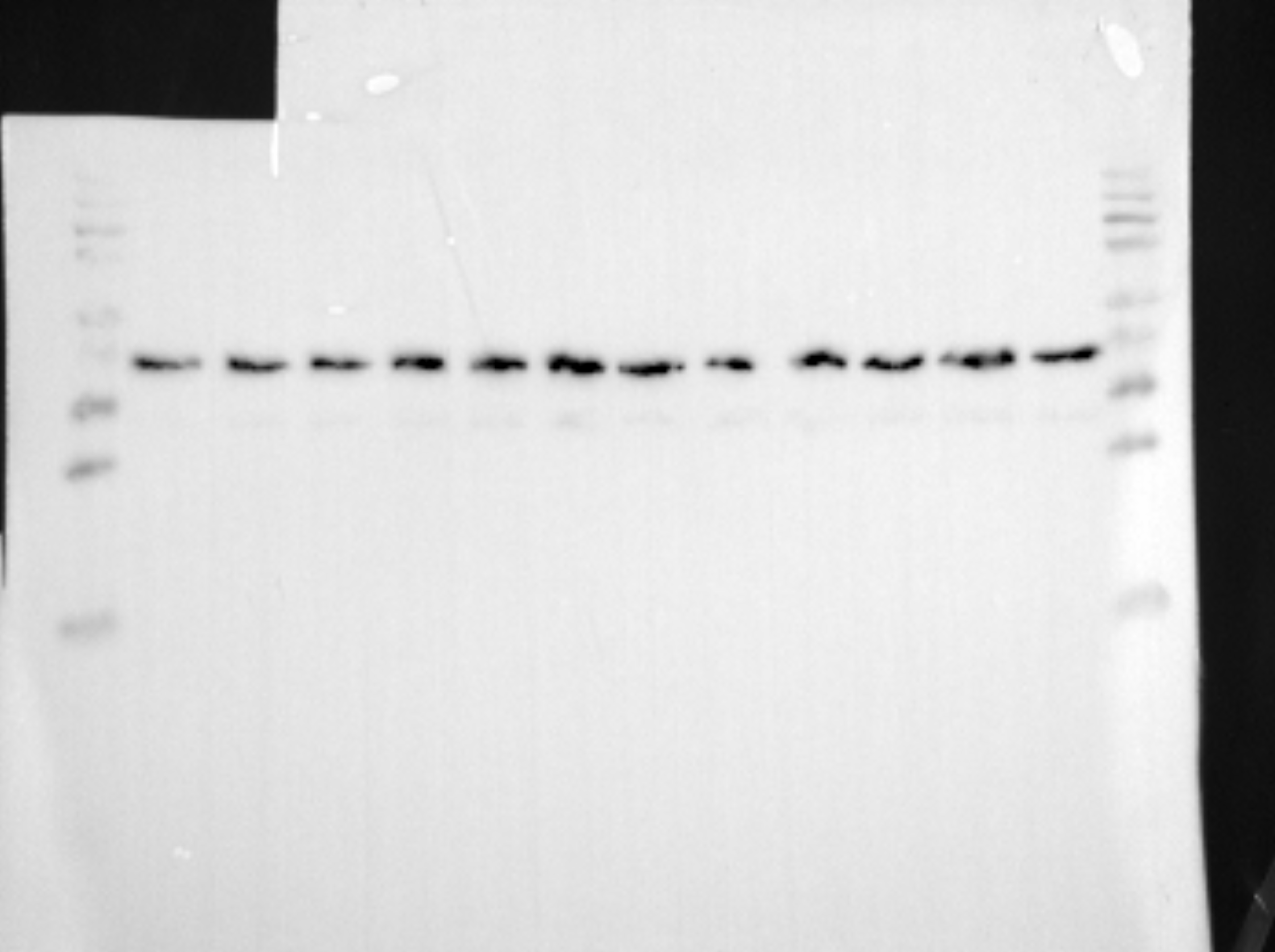

Supplement: Figure 3—source data 1. [file elife-100705-fig3-data1.zip › Phospho-P38.png]
