# Supplementary material for: Multi-omics analyses and machine learning prediction of oviductal responses in the presence of gametes and embryos
Source: eLife. 2025 Feb 26;13:RP100705. doi: 10.7554/eLife.100705 (PMC11864756; doi:10.7554/eLife.100705)
Supplement: Figure 3—source data 2. [file elife-100705-fig3-data2.zip › Figure 3 - source data 2.pdf]

Figure 3 - source data 2

Phospho p38

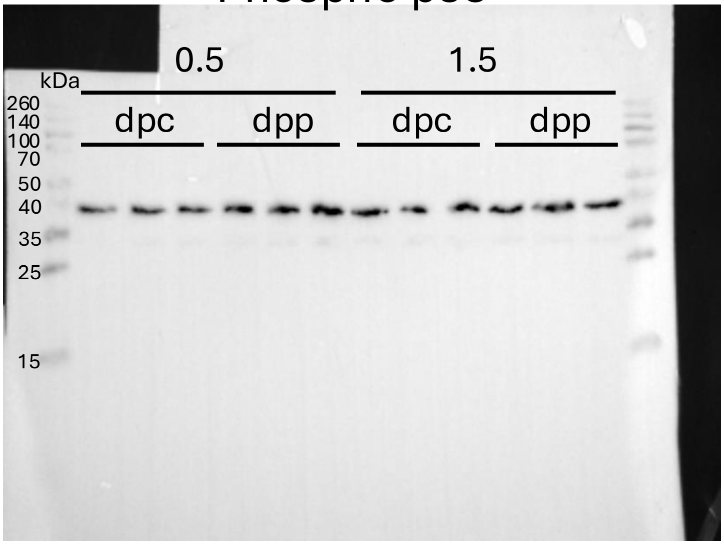

Total p38

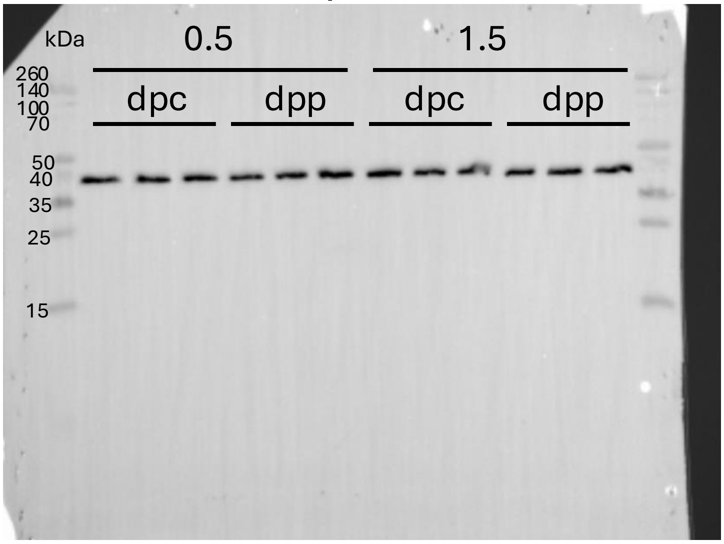

Figure 3 - source data 2: Immunoblotting of phosphorylated p38 and total p38 in the whole oviduct collected at 0.5 dpc, 0.5 dpp, 1.5 dpc, and 1.5 dpp. Each lane represents protein samples from each mouse. A protein ladder was used to determine molecular weights (Spectra™ Multicolor Broad Range Protein Ladder, ThermoFisher, #26634). (n=3 mice/group/timepoint)
