# Supplementary material for: Multi-omics analyses and machine learning prediction of oviductal responses in the presence of gametes and embryos
Source: eLife. 2025 Feb 26;13:RP100705. doi: 10.7554/eLife.100705 (PMC11864756; doi:10.7554/eLife.100705)
Supplement: Supplementary file 6. — (A) scRNA-seq output for each dataset in this study. (B) The result of predicting protein abundance from bulk RNA-seq data at 2.5 dpc by the transformer model. The model is used to classify proteins into two categories, abundant or not abundant, according to two different thresholds: 0.6 and 0.8, respectively. The model can rather accurately predict the abundant proteins from RNA-seq data. [file elife-100705-supp6.pdf]

Supplementary File 6

Multi-omics analyses and machine learning prediction of oviductal responses in the presence of gametes and embryos

Ryan M. Finnerty[1], Daniel J. Carulli[2], Akshata Hegde[3], Yanli Wang[3], Frimpong Baodu[3], Sarayut Winuthayanon[2], Jianlin Cheng[3], and Wipawee Winuthayanon[1,2,*]

Supplementary file 6a: scRNA-seq output for each dataset in this study

Sample Name	Estimate number of cells	Total reads	Mean reads/cell	Median gene/cell	Median UMI counts/cell	Total detected gene #	Sequencing saturation
Ctrl_IA	8,793	89M	10,153	1,387	2,976	19,843	32.1%
Ctrl_IU	10,387	95M	9,173	967	2,194	19,285	36.6%
0.5_IA	3,689	105M	28,382	1,585	3,891	19,044	64.4%
0.5_IU	7,551	93M	12,312	731	1,562	18,612	67.1%
1.5_IA	11,760	84M	7,184	1,138	2,116	19,941	28.0%
1.5_IU	10,480	84M	7,989	860	1,792	19,525	35.6%
2.5_IA	4,504	107M	23,870	1,616	3,590	19,250	60.5%
2.5_IU	7,804	100M	12,871	928	2,178	18,350	65.0%

Supplementary file 6b: The result of predicting protein abundance from bulk RNA-seq data at 2.5 dpc by the transformer model. The model is used to classify proteins into two categories, abundant or not abundant, according to two different thresholds: 0.6 and 0.8, respectively. The model can rather accurately predict the abundant proteins from RNA-seq data.

Protein Abundance Threshold (log-min-max normalized)	Accuracy	F1Score	Precision	Recall
0.8	0.907315	0.922326	0.985852	0.866492
0.6	0.983791	0.78453	0.972603	0.657407
